# Efficacy Of N-Acetyl-Cysteine as Adjuvant Therapy for Diabetic Foot Osteomyelitis: An Open-Label Randomized Controlled Trial

**DOI:** 10.34172/aim.33355

**Published:** 2025-05-01

**Authors:** Laya Hooshmand Gharabagh, Mehdi Heydaroghli, Ayda Esmaeili

**Affiliations:** ^1^Department of Internal Medicine, School of Medicine, Urmia University of Medical Sciences, Imam Khomeini Hospital, Urmia, Iran; ^2^Student Research Committee, Urmia University of Medical Sciences, Urmia, Iran; ^3^Department of Clinical Pharmacy, School of Pharmacy, Urmia University of Medical Sciences, Urmia, Iran; ^4^Patient Safety Research Center, Clinical Research Institute, Urmia University of Medical Sciences, Urmia, Iran

**Keywords:** Inflammatory markers, Diabetic foot osteomyelitis, N-acetyl cysteine, Optimizing treatment response

## Abstract

**Background:**

Biofilm formation by bacteria on the lower limb arises from reduced peripheral arterial blood flow, which can lead to the failure of antibiotic therapy or require longer duration of intravenous antibiotic therapy in diabetic foot infection-associated osteomyelitis. N-acetyl cysteine (NAC), an agent known to prevent and treat biofilm-related infections, was used as a novel strategies beside antibiotic therapy in osteomyelitis of diabetic foot with the aim of accelerating the response to antibiotic therapy regimen.

**Methods:**

To assess the synergistic effect of NAC with antibiotic therapy, patients with diabetic foot osteomyelitis (DFO) (grade III or IV Wagner) were randomly assigned to either NAC 600 mg effervescent tablet twice daily for 2 weeks or the control group. Clinical and laboratory data, including white blood cell with differentiation and inflammatory factors (ESR and CRP) were measured at baseline (time 0), after one week and after three weeks of initiating the intervention.

**Results:**

Fifty-three eligible patients completed the study. All evaluated infectious-related laboratory parameters showed significant reductions in the NAC group compared to control (*P*<0.05), except for lymphocyte proportion and NLR (P; 0.11 and 0.84, respectively). The drop rate of ESR and CRP were accelerated by NAC compared to the control group (-49.44±6.04 vs -7.17±3.99; -44.43±4.21 vs -14.02±4.05, respectively, *P*<0.05).

**Conclusion:**

In order to accelerate antibiotic responses and the trend of reduction in infectious inflammatory markers during the therapy, oral NAC 600 mg twice daily may be considered in the treatment protocol of patients with DFO.

## Introduction

 Diabetic foot ulcer (DFU) is a devastating complication in diabetic patients with 4%‒10% prevalence, especially in poor glycemic control and geriatric patients. If bone infection is present in DFU (osteomyelitis), it is classified as a Wagner’s grade 3 and above.^1^ Approximately three fifths of DFU are infected.^2^ Osteomyelitis is reported in about 50% and 10‒15% of severe and moderate infected DFUs, respectively.^3^ Diabetic foot osteomyelitis (DFO) is categorized as non-device-related bacterial biofilm infection. The failure of and/or need for prolonged duration of intensive doses of intravenous antibiotic therapy are common in DFO. Impairment of peripheral arteries, especially in the lower limbs and high probability of forming biofilm on lower limb bones have led to continued investigation of new adjuvants to improve response to antibiotic therapy.

 N- acetyl-cysteine (NAC) is a known thiol compound and N-acetylated endogenous amine acid L-cysteine, has a wide range of uses, including as mucolytic agent, and an antidote in acetaminophen toxicity.^4^ The multiple mechanisms of action have been described in literature, including (1) antioxidant function through balancing the redox, (2) neutralizing reactive oxygen species (ROS), (3) reduction of inflammatory cytokine such as IL6, IL1β, and TNF-α, and (4) vasodilation through inducing nitric oxide (NO).^5,6^ Additionally, NAC has shown bactericidal properties against *Staphylococcus aureus*, *Enterococcus faecalis*, and *Corynebacterium ammoniagenes* in studies and prevented biofilm formation of Gram-positive and Gram-negative bacteria in *in-vivo* studies.^7-10^ Besides, NAC shows beneficial effects in dermatology conditions such as wound healing.^11^ Intraperitoneal administration of NAC in diabetic and non-diabetic mice for 5 days increased angiogenesis through vascular endothelial growth factor (VEGF) and resulted in faster wound healing.^12^ However, new studies designed for human populations are needed to confirm its efficacy for this specific purpose.

 Considering the above-mentioned pleiotropic actions of NAC, it is hypothesized that NAC can improve therapy response in diabetic foot infection, accelerating wound healing, inhibiting microbial biofilm formation and shortening the course of antibiotic therapy.

 According to recent clinical studies, serial (at least weekly) measurements of C-reactive protein (CRP), white blood cell count (WBC), neutrophil to lymphocyte ratio (NLR) and erythrocyte sedimentation rate (ESR) during treatment guide us to assess response to intravenous (IV) antibiotic therapy in osteomyelitis and are additionally helpful to determine when to switch over from intravenous to oral therapy.^13-16^

 Therefore, the current study was designed to evaluate the effects of oral NAC on response to antibiotic therapy based on the inflammatory factors including ESR and CRP in patients with grade III and IV DFU according to Wagner’s classification.^1^

## Materials and Methods

###  Study Design 

 This randomized, single-center, open-label clinical trial enrolled all patients with DFO admitted to the endocrinology ward of a major academic hospital affiliated to Urmia Medical Sciences University (UMSU) in Urmia, Iran, between December 1, 2020, and November 30, 2022.

###  Study Participants

####  Inclusion Criteria

 Patients aged 18 years or older with DFO, classified as grade III or IV Wagner, were enrolled in the current study. For all patients, bone involvement was confirmed by magnetic resonance imaging (MRI). Vascular evaluation of the lower limbs was done with color Doppler ultrasonography to rule out any thrombosis or any severe vascular involvement. All patients were evaluated by one plastic surgeon within 1‒2 days of admission to assess any need for surgical debridement or amputation (partial, digital or total). The surgeon decided on debridement and/or amputation based on the intensity of devitalized and necrosis of tissue. Clinical signs including pain, erythema, and edema as signs of inflammation, increase or no changes in purulent exudate, foul odor, and deterioration in the appearance of the wound were assessed by the physician to determine the intensity of antibiotic therapy.

 Participants with a PEDIS score (Perfusion, Extent, Depth, Infection and Sensation) ≥ 7 were eligible to enter the study, which indicates that the patient is high risk and should have intensive antibacterial regimen intervention.^18^

####  Exclusion criteria

 (1) Already being treated with any known supplements that have anti-inflammatory and anti-oxidative effects such as Vitamin C, D, E and A, herbal supplements such as curcumin, etc.; (2) administration of NAC at least 2 weeks before hospitalization; (3) history of hypersensitivity reaction to NAC or sulfur products; (4) Other causes of ulcers besides diabetes including trauma, skin disease, and rheumatologic disease; (5) patients with active malignancy or who received chemotherapy or radiotherapy within the previous year; (6) patients with a Glasgow Coma Scale score of 12 or less and those who could not tolerate oral agents; (7) pregnancy or breastfeeding; (8) patients on immunosuppressant medications such as corticosteroids ( ≥ 40 mg prednisolone equivalent dose), mycophenolate, cyclosporine, tacrolimus, or rituximab, which interfere with wound healing; (9) hospitalization for a DFU in the past month and receiving injection antibiotic treatment; (10) patients with chronic kidney disease with creatinine clearance < 15 mL/min^19^ or undergoing dialysis, and those with decompensated liver disease (considered to be immunocompromised^20^); (11) substance addiction; (12) those requiring foot amputation or needing revascularization or amputation (partial, digital or total), and those with DFU in grade V Wagner.^21^

###  Sample Size and Randomization 

 Based on the mean wound healing score after surgery in a previous study by Oguz et al (31.89 ± 2.26 in the control group and 33.98 ± 2.14 in the N-acetylcysteine ​​group), considering a 95% confidence interval (Z_1-α/2 _= 1.96) and a power of 90% (Z_1-β _= 1.28), we calculated a sample size of 28 patients in each group using the following formula^22^:


$$n=\frac{{\left(z_{1}-\frac{\alpha }{2}+z_{1}-\beta \right)}^{2}\;x(s_{1}^{2}+s_{2}^{2})}{{\left({\bar{x}}_{1}-{\bar{x}}_{2}\right)}^{2}}$$

Eligible participants were randomized using block randomization; given the total sample size of 56 patients, 14 blocks of 4 were used. The randomized list of numbers 1 to 56 was randomly divided into two groups of A (intervention group) and B (control group), and the subjects were alternately allocated into group A or B, yielding 28 people in each group. The intervention group received NAC in oral effervescent form 600 mg twice daily for 14 days, in concurrent use with intravenous antibiotic medications vancomycin or teicoplanin (if allergic to vancomycin), ciprofloxacin and carbapenem (imipenem- cilastatin or meropenem depending on availability). This dosage of NAC was chosen from trials related to pulmonary infections.^23-25^

 According to the DFO treatment guideline, the length of systemic antibiotic therapy was considered to be 3 weeks. The control group only received rational intravenous antibiotic medications. Bacterial culture from the wound and blood were obtained on the day of admission for all patients. The results of culture were used to identify the pathogenic bacteria in wound discharge, and guide appropriate and effective antibiotics. Patients’ demographic data including age, gender, height and weight for calculating body mass index (BMI), number of wounds, onset and duration of foot ulcer, duration of diabetes, and any other medical history were gathered. Laboratory data such as blood glucose, blood urine nitrogen (BUN), serum creatinine, and complete blood cell count and differential were assessed at the beginning and after three weeks. In addition, baseline CRP and ESR levels were measured and rechecked after one week and around the time of discharge (19‒23 days of admission, three weeks). All patients were evaluated by the investigators daily and any possible side effects of NAC were recorded using Naranjo as an Adverse Drug Reaction Probability Scale.^26^ At least 80% adherence to medication was considered as acceptable compliance.^27^

###  Statistical Analysis

 All the statistical analyses were conducted using the SPSS statistics software (version 20.0, SPSS Inc., Chicago, IL, USA). Categorical data was expressed by frequency (percentage). Normally and not normally distributed continuous values were presented as mean ± SD and/or median (interquartile range), respectively. The normality assumption of data was assessed with the Kolmogorov–Smirnov test and Q-Q plot. Then, the means of variables with normal or non-normal distribution were compared between the two groups using parametric or nonparametric tests including independent *t* test/ ANCOVA or Mann-Whitney U test, respectively. For comparing the within-group changes, we used a paired sample *t *test or Wilcoxon Rank test for variables with normal or non-normal distribution, respectively. The per-protocol method was used for data analysis. A *P *value < 0.05 was considered as significant.

## Results

###  Patient Characteristics

**Figure 1:**
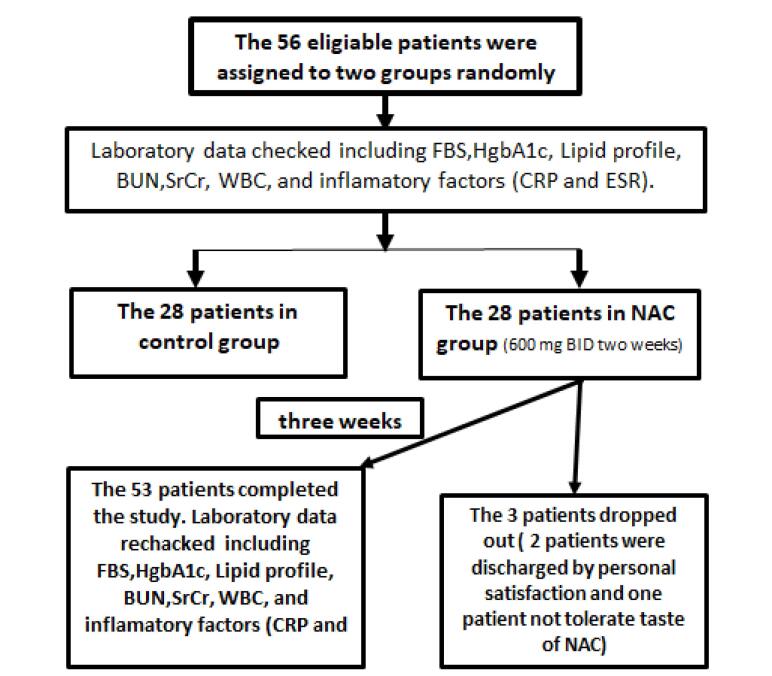
Flow Diagram of Study

 A total of 56 patients with confirmed DFO were enrolled in current study; 28 patients were assigned to each group. Three patients in the NAC group were excluded and the other participants in both groups completed the trial (Figure 1). The patients’ demographic and diagnostic information of DFO is listed in Table 1. The patients who completed the study improved clinically over the hospitalization course and were discharged with acceptable clinical status on oral antibiotics agents. There was no statistically significant difference between the groups regarding their characteristics except for age (NAC group: 68.00 ± 13.78, control group: 57.74 ± 8.32; *P* value = 0.003).

**Table 1:** Patients; Characteristics at Baseline

**Group (n=60)**	**NAC Group (n=25)**	**Control Group (n=28)**	***P *** Value^*^
Age, year, mean ± SD	68.00 ± 13.78	57.74 ± 8.32	0.003
Sex (F/M)	17/8	16/12	0.42
BMI (kg/m^2^)	26.37 ± 2.38	27.88 ± 2.75	0.06
Wagner classification grade of DFO (grade 3/grade 4)	11/14	18/10	0.55
Ulcer duration (months)	1.75 ± 0.96	2.21 ± 0.63	0.12
Number of DFU			
1	11	18	0.55
> 1	14	10	
Regimen of antibiotic therapy			
Vancomycin + meropenem	10	15	
Vancomycin + ciprofloxacin + meropenem	11	9	
Others	4	3	
Microbiologic results of wound swab culture			
Negative	12	8	
Positive	13	20	

DFO, Diabetic foot osteomyelitis; BMI, Body Mass Index; DFU, Diabetic foot ulcer; n, Number of patients. * *P* value < 0.05 is significant.

 According to our findings, contrary to the control group, WBC and the N/L ratio significantly improved over three weeks’ therapy compared to baseline value in the NAC group (WBC at the end of study in NAC group: 7807.80 ± 2720.48 vs. control group: 8530 ± 2775.89; Neutrophil ratio in NAC group: 63.78% ± 8.38% vs. control group: 74.32% ± 8.77%; *P*< 0.01). However, there was no statistically significant difference in the N/L ratio changes between the two groups (N/L ratio change in NAC group: 7.25 ± 3.99 to 5.12 ± 2.23 vs. control group: 10.34 ± 9.17 to 8.5 ± 5.19; *P* value = 0.84; Table2).

**Table 2:** Comparing Laboratory Data at Baseline and End of Study in NAC and Control Groups

	**Patients NAC 600 mg BID for 2 weeks (n=25)**			**Control Group (n=28)**			***P*** ** Value Between Groups at Baseline**	***P*** ** Value of Changes of Variables Between Groups at End**
	**Baseline**	**End**	***P*** ** Value**	**Baseline**	**End**	***P*** ** Value**		
Hgb-A1C (%)	9.53 ± 1.91	9.13 ± 1.99	0.001	9.44 ± 1.04	9.44 ± 1.04	0.63	0.33	0.001*
FBS (mg/dL) ^#^	219.48 ± 84.67	152.36 ± 29.75	< 0.001	179.85 ± 38.8	162.61 ± 28.93	0.006	0.04	0.004^¥^
WBC (per microliter)	10223.36 ± 3039.70	7807.80 ± 2720.48	0.001	9002.50 ± 2289.6	8530 ± 2775.89	0.22	0.10	0.01*
Neutrophil %	75.72 ± 8.38	63.78 ± 8.38	< 0.001	78.32 ± 8.60	74.32 ± 8.77	0.001	0.27	< 0.001*
Lymphocyte %	12.96 ± 5.46	14.96 ± 6.87	0.02	11.18 ± 6.08	14.66 ± 6.40	0.32	0.26	0.11*
N/L ratio	7.25 ± 3.99	5.12 ± 2.23	0.003	10.34 ± 9.17	8.5 ± 5.19	0.14	0.13	0.84*

Hgb-A1C, Hemoglobin A1C; FBS, Fast blood sugar; N, Neutrophil; NLR, Neutrophil / Lymphocyte ratio; n, Number of patients. * Comparison made with independent T-test. ^¥^ Comparison made with ANCOVA test. ^#^ Comparison made with Mann-Whitney U test. *P* value < 0.05 is significant.

**Figure 2:**
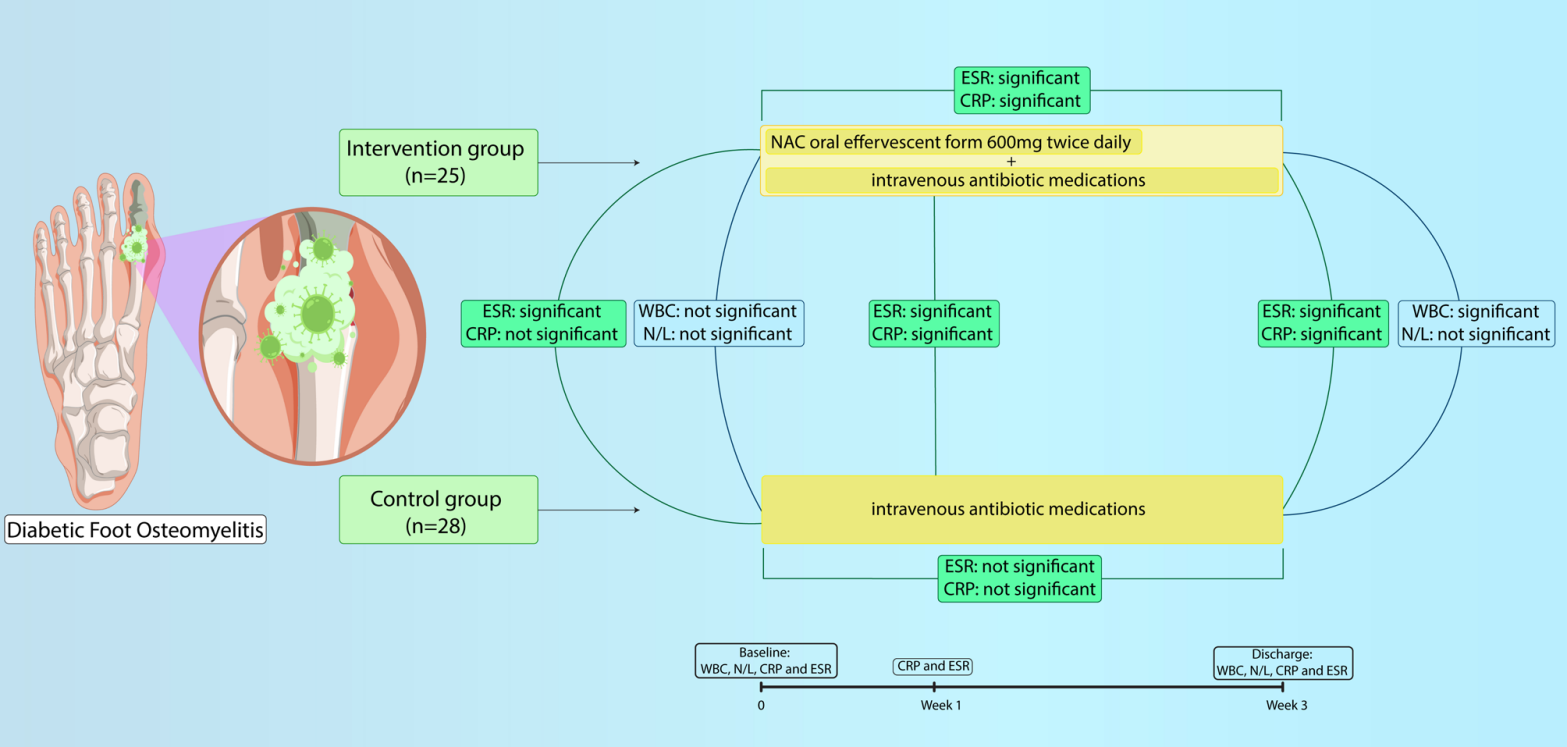
Study process and results illustration

Table 3 compares the assessed inflammatory variables including ESR and CRP at baseline, after one week and at discharge time (one week after the end of intervention) in each group and within groups. According to analysis, both ESR and CRP in patients on NAC decreased significantly after one week and at discharge time (ESR at baseline: 87.08 ± 28.90 vs. after a week: 76.48 ± 26.24 vs. at discharge: 37.64 ± 22.36; *P* value < 0.001) (CRP at baseline: 73.57 ± 26.53 vs. after a week: 61.14 ± 25.22 vs. at discharge: 29.13 ± 21.05; *P* < 0.001), while in the control group, non-significant changes were observed in ESR and CRP after one week of starting only systemic antibiotics (*P* = 0.09 and 0.05, respectively) and the rate of decline in ESR and CRP level over the length of admission were slower (Figure 2).

**Table 3:** Comparing Inflammatory Markers at Baseline and Hospital Discharge in NAC and Control Groups

		**NAC Group**	**Mean of Changes**	***P *** Value^*^	**Control Group**	**Mean of Changes**	***P *** Value^*^	***P *** **Value**^¥^** Between Groups**
		**Mean±SD**	**Mean±SE**		**Mean±SD**	**Mean±SE**		
ESR	Baseline	87.08 ± 28.90		‒	54.07 ± 19.51			< 0.001
	After one week of starting intervention	76.48 ± 26.24	-10.59 ± 2.41	< 0.001	52.53 ± 18.24	-1.53 ± 0.87	0.09	0.001
	Hospital discharge (after 3 weeks)	37.64 ± 22.36	-49.44 ± 6.04	< 0.001	46.89 ± 21.87	-7.17 ± 3.99	0.08	
CRP	Baseline	73.57 ± 26.53			63.97 ± 27.73			0.18
	After one week of starting intervention	61.14 ± 25.22	-12.43 ± 2.41	< 0.001	61.14 ± 25.22	-2.11 ± 0.51	0.05	< 0.001
	Hospital discharge (after 3 weeks)	29.13 ± 21.05	-44.43 ± 4.21	< 0.001	49.94 ± 30.59	-14.02 ± 4.05	0.002	

NAC, n-acetyl cysteine; ESR, Erythrocyte sedimentation rate; CRP, C-reactive protein; n, number of patients. ^*^ *P* value of comparison within group. ^¥^ *P* value of comparison between groups. *P* value < 0.05 is significant.

 One patient stopped receiving therapy due to the unpleasant taste of effervescent NAC. Also, two patients in the NAC group dropped out of study due to early discharge (personal consent) and not completing the duration of intervention.

## Discussion

 As an antioxidant agent with pleiotropic effects including antibacterial property simultaneously with inhibiting biofilm formation, NAC is used as an adjuvant therapy to accelerate cure in various infections such as pneumonia.^23,28^ To the best of our knowledge, there is no published clinical study in which NAC was used as an adjuvant therapy in DFO. Therefore, the current study was designed to evaluate the effect of NAC on the outcome of DFO antibiotic therapy. According to our findings, NAC substantially accelerated the rate of decline in ESR, CRP, WBC count, and N/L ratio which are used to evaluate response to antibiotic therapy.

 The normal ranges of WBC count and NLR in the healthy population are 4500 to 11 000/m^3^ and 0.78 to 3.5, respectively.^29^ Neutrophils play a major role in regulating innate immunity, through activating other immune cells and secreting pro inflammatory cytokines which stimulate the functions of other immune cells such as dendritic cell, T and B cell lymphocytes in infections and inflammatory diseases.^30^ The higher NLR value and neutrophil-dominant WBC counts are proven to have independent direct correlation with morbidity and mortality rate in various diseases including infections.^31^ Leukocytosis and increase in NLR are valuable biomarkers for early diagnosis of infectious diseases including DFO as well as predictors for needing long-term intravenous (IV) antibiotic and hospitalization.^32,33^ Altay et al showed that the decrease in NLR during the first 14 days of starting treatment had a direct correlation with improvement in DFU and appropriate response to therapy IV antibiotic therapy.^34^ Therefore, they are reliable indicators for monitoring response to antibiotic therapy and healing of the foot ulcer.

 Our study showed that patients on NAC experienced a significant reduction in WBC count and neutrophil percentage compared to the control group who received only antibiotic therapy, while there was no statistically difference in NLR value changes. Nevertheless, the decrease in NLR over two weeks in the intervention group was significant, while in the control group, it was not.

 Serum inflammatory markers, including CRP, ESR, and WBC are known as diagnostic markers for diabetic foot bone infection with acceptable sensitivity and specificity (above 0.70).^35^ Optimal cut-off points for ESR and CRP levels as diagnostic markers have not been defined so far.^36,37^ However, according to Lavery et al, an ESR level more than 60 mm/h and a CRP level greater than 7.9 mg/dL (with statistically acceptable specificity and sensitivity) are optimal cut-off points for predicting osteomyelitis and the physician should consider osteomyelitis treatment for these patients.^13^ It is worth noting that, the CRP, WBC count, and NLR simultaneously (faster than ESR) start to decline during the therapy and rapidly help us to assess the treatment response.^38^ For instance, an original article by van Asten et al on 122 patients with DFO showed a direct correlation between reduction in ESR and CRP levels during therapy and acceptable clinical outcomes.^38^ Van Asten et al conducted another study to evaluate the value of inflammatory markers in monitoring treatment response in 35 patients hospitalized with FDO; they reported that CRP, ESR, procalcitonin, and interleukin-6 are valuable markers for assessing response to antibiotic therapy.^39^

 Considering the value of CRP and ESR levels in treatment monitoring, patients who received NAC for 14 days had significantly lower levels of both serum inflammatory markers, CRP and ESR, on discharge than the control group. Therefore, it is concluded that NAC has a synergistic effect in combination with antibiotic therapy. The decrease rate of CRP and ESR in patients on NAC was approximately three and seven times faster in comparison with the control group. Additionally, after a week, CRP and ESR changes in the control group were not significant, meanwhile for patients on NAC, a dramatic reduction was seen even after a week. According to the results of Durak et al, CRP is the superior marker over ESR for evaluating early therapeutic response of antibiotics.^40^

 Hence, it can be concluded that NAC might be a potent adjuvant agent beside antibiotics to accelerate response and shorten the duration of intravenous therapy; it is associated with early hospital discharge and improved patients’ compliance for the approximate 3 months of antibiotic therapy.

## Limitation

 This study had some limitations as follows: (1) The sample size in the current study was small which may have affected the outcome. With a larger sample size, the results can be different and the power of study is increased. (2) It is better to follow up the patients for a month and until the end of the total duration of antibiotic therapy (IV and oral; three months), and for a year to evaluate the rate of recurrence of DFO and need for readmission, which may result in decreasing the financial burden on patients with DFO, who need a longer length of stay in hospitals. (3) All patients clinically improved over 3 weeks receiving antibiotic therapy but we did not report the time to recovery of clinical features; it is recommended to evaluate the recovery time of these clinical signs, as well.

###  Recommendation

 Based on our results, NAC is a promising candidate for accelerating inflammatory factors’ response to antibiotic therapy which may result in shortening the long antibiotic therapy; therefore, it is recommended to conduct further studies on NAC as an adjuvant therapy with antibiotics with various aims of reducing the duration of IV antibiotic therapy, length of hospital stay and recurrence of DFO over one year.

## Conclusion

 Oral NAC 600 mg BID along with antibiotic therapy significantly reduced the inflammatory markers of therapeutic response, including CRP and ESR. Based on this evidence, NAC may be a proper option to use as an adjutant agent in the treatment protocol of DFO with the aim of early switching from IV to oral therapy and shortening the length of hospitalization.
